# Different types of human–AI interaction and employees’ knowledge territorial behavior: the role of organizational dehumanization and paradoxical leadership

**DOI:** 10.3389/fpsyg.2026.1854239

**Published:** 2026-07-29

**Authors:** Ziyi Zhang, Yuying Wu

**Affiliations:** 1 School of Economics and Management, Shihezi University, Shihezi, China; 2 School of Law, Shihezi University, Shihezi, China

**Keywords:** human–AI interaction, enhanced human–AI interaction, impeded human–AI interaction, knowledge territoriality, organizational dehumanization, paradoxical leadership

## Abstract

**Introduction:**

As artificial intelligence (AI) becomes increasingly embedded in organizational workflows, understanding how human–AI interactions shape employees’ knowledge territorial behavior has become a critical issue in digital transformation. Drawing on conservation of resources theory, this study examines the differential effects of enhanced and impeded human–AI interaction on employees’ knowledge territorial behavior, with organizational dehumanization as the underlying psychological mechanism and paradoxical leadership as a boundary condition.

**Methods:**

We tested the hypotheses across two studies. Study 1 used a scenario-based experiment to establish the causal effects of different forms of human–AI interaction. Study 2 employed a two-wave field survey to validate the full moderated mediation model in a real-world organizational context.

**Results:**

Enhanced human–AI interaction was negatively associated with knowledge territorial behavior, whereas impeded human–AI interaction was positively associated with such behavior. Organizational dehumanization significantly mediated these relationships. In addition, paradoxical leadership moderated these effects: higher levels of paradoxical leadership strengthened the negative effect of enhanced human–AI interaction on organizational dehumanization and attenuated the positive effect of impeded human–AI interaction on organizational dehumanization.

**Discussion:**

These findings reveal the double-edged effects of human–AI interaction on employees’ knowledge territorial behavior. This research extends the literature on the antecedents of knowledge territoriality, highlights organizational dehumanization as a key psychological mechanism, and identifies paradoxical leadership as an effective managerial approach for facilitating human–AI collaboration in modern organizations.

## 1 Introduction

Knowledge territorial behavior arises from individuals’ perceived ownership of specific knowledge and is reflected in behaviors aimed at asserting and protecting knowledge boundaries, such as knowledge marking (e.g., claiming ownership) and knowledge defense (e.g., encrypting information or refusing to share it) (Brown et al., 2014a,b; Tan et al., 2024; Xu and Li, 2022). As artificial intelligence (AI) becomes deeply embedded in organizational settings, both the nature of work and the logic of resource allocation are being fundamentally reconfigured. Although AI’s superior computational and generative capabilities have enhanced efficiency, they have also made it easier to transform tacit knowledge into explicit knowledge, thereby exposing employees to a heightened perceived threat that their knowledge may be appropriated, devalued, or even rendered substitutable. Recent evidence suggests that this concern is not merely theoretical. A TechRadar report, drawing on survey findings from Adaptavist, indicates that 35% of employees deliberately engage in “gatekeeping” to reduce the risk of being replaced by AI, while 38% refuse to train colleagues in areas of personal expertise (Hale, 2025). Such defensive responses, aimed at buffering against AI-related disruption and restoring psychological security, have increasingly become a hidden barrier to digital transformation. Given the detrimental consequences of knowledge territorial behavior for knowledge flows and organizational innovation, examining whether and how AI involvement in work may trigger or suppress such behavior has become a pressing issue for both organizational scholarship and managerial practice.

Existing research, drawing primarily on social cognitive theory, conservation of resources (COR) theory, and psychological ownership theory, has consistently identified negative leadership styles (e.g., bottom-line mentality), low organizational trust, and individual-level factors as key antecedents of employees’ knowledge territorial behavior (Brown et al., 2005; Brown et al., 2014a,b; Brown and Robinson, 2011; Greenbaum et al., 2012; Mesdaghinia et al., 2019; Silva de Garcia et al., 2022; Zhang et al., 2020). However, these studies have largely focused on traditional interpersonal and organizational contexts. Amid digital transformation, the growing presence of algorithms and intelligent technologies has disrupted conventional resource boundaries in the workplace. AI is not merely a tool for information provision; it is increasingly also a nonhuman actor that reshapes work design and participates in decision-making processes (Aydin and Karaarslan, 2023; Bailey et al., 2022; Parker and Grote, 2022; Raisch and Krakowski, 2020; Yam et al., 2023). Although scholars have begun to examine the implications of AI for employee psychology and behavior, little is known about how different forms of human–AI interaction may reconfigure employees’ psychological resource states and, in turn, produce either inhibitory or facilitative effects on knowledge territorial behavior. Addressing this question is critical for understanding and mitigating knowledge territoriality in the digital era, while also promoting organizational performance and innovation.

To address this gap, the present study draws on COR theory and introduces organizational dehumanization as a key psychological mechanism linking human–AI interaction to knowledge territorial behavior. Following Li et al. (2025), human–AI interaction can be distinguished into enhanced and impeded forms. However, we clarify that this distinction should not be understood as being determined solely by the allocation of control between employees and AI. Rather, enhanced versus impeded human–AI interaction captures employees’ subjective evaluation of whether AI involvement facilitates or obstructs task completion, workflow efficiency, and the preservation of valued work resources. Control allocation remains an important feature of human–AI interaction, but it is better understood as one mechanism through which AI may become enhancing or impeding, rather than as the exclusive criterion for distinguishing the two forms. Enhanced human–AI interaction refers to situations in which employees perceive AI as supporting their work, reducing unnecessary burdens, and improving their capacity to complete tasks effectively. By contrast, impeded human–AI interaction refers to situations in which employees perceive AI as disrupting workflows, increasing adaptation pressure, or constraining effective task accomplishment. In this sense, an interaction characterized by high AI autonomy may still be impeded if the technology is inefficient, intrusive, or poorly aligned with employees’ work needs. Conversely, an algorithm-dominated process may be experienced as enhanced when it accelerates workflow, reduces employees’ cognitive burden, and improves task outcomes. We argue that organizational dehumanization, defined as an individual’s subjective perception that they are being objectified or treated merely as an instrument by the organization, serves as a critical explanatory bridge in this process (Baldissarri and Fourie, 2023; Bell and Khoury, 2011). Importantly, although employees interact directly with a nonhuman technology, the AI system is not experienced as an isolated technological artifact. Rather, it is introduced, configured, authorized, and embedded into work routines by the organization (Jiang et al., 2026; Chen et al., 2026; Zoppelletto et al., 2026; Li et al., 2026). Thus, when an algorithm assigns tasks, monitors workflows, or constrains employees’ discretion, employees may interpret these algorithmic arrangements as reflecting the organization’s preferred way of managing human labor. In this sense, the immediate stressor may be technological or algorithmic, but its social meaning is organizational because the technology operates as an organizationally sanctioned mechanism of coordination and control. From a COR perspective, enhanced interaction constitutes a resource-preserving process that helps employees maintain valued psychological resources such as control, autonomy, and professional efficacy, thereby reducing organizational dehumanization. By contrast, impeded interaction operates as a persistent environmental stressor: algorithmic control deprives employees of autonomy and a sense of agency, intensifies resource depletion, and increases feelings of objectification (Deci and Ryan, 2000; Lammers and Stapel, 2011). When employees perceive that the organization relies on AI primarily to standardize, monitor, and optimize their labor while neglecting their judgment, dignity, and human uniqueness, they are more likely to conclude that the organization treats them as replaceable instruments for achieving organizational goals. Faced with resource loss and dehumanization threats, employees are likely to adopt defensive strategies to prevent further depletion. Knowledge territorial behavior thus functions as a resource-protection response through which employees monopolize core knowledge in order to preserve their irreplaceability, restore control, and defend their workplace standing (Stinglhamber et al., 2025). Taken together, organizational dehumanization is expected to mediate the relationship between human–AI interaction and knowledge territorial behavior.

Moreover, the effects of human–AI interaction on employees’ psychological resources are likely to depend on the broader leadership context. Human–AI collaboration inherently embodies the tension between standardized algorithmic logic, which emphasizes efficiency, and human value logic, which emphasizes dignity and individuality. As a leadership approach oriented toward simultaneously accommodating competing demands, paradoxical leadership is particularly well-suited to managing this tension (Zhang, 2015). Consistent with COR theory, paradoxical leadership can be understood as an important contextual resource that provides dynamic resource support by balancing contradictory demands (Zhang, 2015; Zhang et al., 2022). In contexts of enhanced interaction, high levels of paradoxical leadership may facilitate the integration of human and technological strengths, broaden employees’ channels of resource acquisition, and strengthen the resource-preserving effect of enhanced interaction (Avolio et al., 2004), thereby amplifying the negative association between enhanced interaction and organizational dehumanization. By contrast, in contexts of hindering interaction, the inclusiveness and individualized consideration inherent in paradoxical leadership may compensate for the psychological resource losses caused by algorithmic control, helping employees regain dignity and autonomy within system constraints and thereby weakening the positive effect of impeded interaction on organizational dehumanization (Vaughn, 2017; Volk et al., 2023).

This study makes three primary contributions. First, it extends the literature on the antecedents of knowledge territorial behavior by incorporating a human–AI interaction perspective. Prior research has largely focused on traditional individual and contextual antecedents (Silva de Garcia et al., 2022; Tims and Bakker, 2010), whereas this study shifts attention to digitally mediated work settings and reveals the double-edged effects of enhanced and impeded human–AI interaction on employees’ knowledge territorial behavior (Li et al., 2025). In doing so, it not only captures the practical reality that employees may deliberately withhold core expertise out of fear of AI-driven replacement, but also clarifies the theoretical conditions under which algorithmic dominance erodes psychological security and triggers territorial responses. Second, by drawing on COR theory, this study uncovers the psychological mechanism through which human–AI interaction shapes knowledge territoriality. Specifically, it introduces organizational dehumanization as a key mediator, thereby enriching research on the psychological consequences of human–AI collaboration and explaining why employees may resort to knowledge territorial behavior as a defensive strategy to cope with dehumanization threats and restore resource equilibrium. Third, this study extends research on paradoxical leadership. Although prior work suggests that employees under paradoxical leadership may be more likely to leverage AI for knowledge sharing (Hu et al., 2025), little attention has been paid to whether such leadership can mitigate the adverse effects associated with AI-induced resource loss and dehumanization. Our framework suggests that paradoxical leadership can alleviate employees’ organizational dehumanization and thereby reduce knowledge territorial behavior. This insight not only advances understanding of the boundary conditions for the effectiveness of paradoxical leadership, but also offers practical implications for leadership interventions aimed at curbing knowledge territorial behavior at its source during digital transformation.

## 2 Theoretical basis and research assumptions

### 2.1 Human–AI interaction

The present study’s conceptualization of human–AI interaction is directly anchored in the framework developed by Li et al. (2025). Operating strictly at the individual level, this taxonomy distinguishes between two distinct forms of subjective interaction experiences based on differences in control allocation and workplace dominance: enhanced human–AI interaction and impeded human–AI interaction. We further clarify that the enhanced–impeded distinction does not simply reflect who controls the work process. Although control allocation and workplace dominance are important features of human–AI collaboration, the core distinction lies in whether employees perceive AI involvement as facilitating or obstructing task completion, workflow efficiency, and resource preservation. Thus, control allocation is treated as a salient manifestation and potential antecedent of different interaction experiences, rather than as the sole basis for classification. Enhanced human–AI interaction refers to the extent to which employees perceive AI as supporting their work, improving efficiency, reducing repetitive burdens, and strengthening their ability to complete tasks effectively (Li et al., 2025). In this supportive configuration, AI typically takes over labor-intensive or repetitive tasks, such as data processing and information sorting, whereas employees retain ultimate responsibility for core judgment and decision-making. Importantly, enhanced interaction does not necessarily require employees to retain full control over every work procedure. When algorithmic systems accelerate workflow, reduce cognitive burden, and improve task outcomes, employees may still experience the interaction as enhancing even if AI plays a substantial role in the work process (Tang et al., 2022; Wang et al., 2023; Siemsen et al., 2008). By contrast, impeded human–AI interaction refers to the extent to which employees perceive AI as obstructing their work, increasing adaptation pressure, disrupting established workflows, or constraining their ability to accomplish tasks effectively. In such restrictive configurations, algorithms frequently dictate task allocation, monitor workflows, and even override human decision outcomes, leaving employees primarily responsible for mechanical execution. Nevertheless, algorithmic dominance is not by itself equivalent to impeded interaction. A human–AI interaction becomes impeding when AI involvement undermines employees’ work effectiveness, autonomy, or resource states, such as when the technology is inefficient, intrusive, inaccurate, or poorly aligned with employees’ actual work needs. Therefore, our conceptualization preserves the insight of Li et al. (2025) while more precisely distinguishing between control allocation as a structural feature and enhanced versus impeded interaction as employees’ perceived functional experience of AI involvement (Koo et al., 2021; Rinta-Kahila et al., 2023).

### 2.2 Knowledge territorial behavior

Knowledge territorial behavior refers to a set of behavioral manifestations through which individuals construct, claim, maintain, and restore control over specific knowledge in the workplace (Peng, 2013). As discussed earlier, this construct is primarily manifested in two forms of behavior: knowledge marking and knowledge defense (Tan et al., 2024). Existing research has shown that employees are more likely to engage in such behaviors when they face low-trust environments, intense competitive pressure, or threats to their psychological ownership. These antecedents include leaders’ bottom-line mentality (Tan et al., 2024; Zhang et al., 2020), expectations of betrayal (Gardner et al., 2019), moral disengagement (Tan et al., 2024), psychological ownership (Brown et al., 2005; Silva de Garcia et al., 2022), perceived unfairness (Brown and Robinson, 2011), low organizational trust (Brown et al., 2014a,b), employees’ activated prevention focus (Peng, 2013; Xu and Li, 2022), and cost–benefit considerations in resource regulation (Demerouti et al., 2001). Collectively, these studies provide an important theoretical foundation for understanding employees’ territorial responses related to knowledge.

Nevertheless, research on knowledge territorial behavior remains at an early stage. On the one hand, because this behavior is highly sensitive to interactional contexts and psychological cues, it offers a particularly direct window into how employees manage knowledge under conditions of resource competition and relational uncertainty. Yet extant work has focused predominantly on traditional work settings, with limited attention to how digitally mediated interactions, particularly human–AI interaction, may shape such behavior. This omission makes it difficult to fully capture the evolving nature of employees’ knowledge territorial responses in the digital era. On the other hand, the mediating and moderating mechanisms underlying knowledge territorial behavior remain insufficiently examined, which constrains both scholarly understanding and practical intervention. Accordingly, investigating how human–AI interaction may trigger or suppress knowledge territorial behavior is critical for addressing an important gap in contemporary knowledge management research.

### 2.3 Paradoxical leadership

Paradoxical leadership refers to a leadership style in which leaders integrate seemingly contradictory yet interdependent behavioral demands, such as maintaining control while granting autonomy and enforcing consistency while allowing individualization, in order to respond effectively to complex organizational environments (Zhang, 2015). In the AI-enabled workplace, human–AI collaboration inherently embodies an important tension between two competing logics. On the one hand, algorithmic technologies introduce an instrumental rationality characterized by the pursuit of maximum efficiency and standardized control; on the other hand, employees continue to value a human-centered rationality grounded in the preservation of subjective agency, dignity, and interpersonal needs (Park et al., 2024; Tang et al., 2023a,b). Importantly, the effects of this tension are not fixed but instead depend on contextual conditions (Wijayati et al., 2022). Among various leadership styles, paradoxical leadership is particularly relevant because it emphasizes the simultaneous accommodation of competing demands and the balancing of performance expectations with humanistic concern (Volk et al., 2023; Zhang et al., 2015). However, we do not assume that paradoxical leadership automatically buffers the negative consequences of AI-based control. If leaders merely add another layer of contradictory control to an already over-controlling algorithmic system, employees may indeed experience greater role ambiguity and cognitive overload. The buffering role of paradoxical leadership depends on whether leaders can integrate, rather than simply accumulate, competing demands. In this study, paradoxical leadership is conceptualized as a contextual resource because high levels of such leadership provide employees with clearer explanations, role clarification, individualized concern, and discretion within system constraints (Zhang et al., 2015). This feature makes it especially compatible with human–AI interaction contexts, where efficiency-oriented logic and human-centered needs coexist. From the perspective of conservation of resources (COR) theory, paradoxical leadership can be understood as a critical contextual resource that helps regulate employees’ resource preservation and resource loss in the face of technological disruption. Specifically, in contexts of hindering interaction, paradoxical leadership may provide dignity, support, and individualized care, thereby buffering the psychological resource depletion associated with algorithmic control. In contexts of enhanced interaction, it may help employees better integrate technological benefits into their work, thereby reinforcing the resource-preserving effects of AI support. Incorporating paradoxical leadership into the theoretical framework of human–AI interaction therefore moves beyond the conventional tendency to treat leadership merely as a direct antecedent variable (Yang et al., 2021a,b; Zhang and Jiang, 2015). More importantly, it helps clarify the managerial conditions under which organizations can effectively mitigate the dehumanizing potential of algorithms or, alternatively, strengthen their empowering effects, thus identifying a critical boundary condition for understanding and reducing employees’ knowledge territorial behavior.

## 3 Theoretical background and hypotheses

### 3.1 Theoretical background

Conservation of resources (COR) theory provides the core theoretical foundation for understanding how employees’ psychological states and behavioral responses evolve in digitally transformed work contexts. COR theory posits that individuals are strongly motivated to acquire, protect, and retain resources they value (Halbesleben et al., 2014). In contemporary organizations, such resources extend beyond tangible job conditions to include critical psychological resources, such as control over work processes, professional dignity, and a sense of subjective agency (Hobfoll et al., 2018). Although COR theory discusses resource gain spirals, resource loss spirals, and resource defense principles, the present study does not directly test a full resource gain spiral. A gain spiral would require empirical evidence that an initial resource gain enables the subsequent accumulation of additional positive resources over time. Because our mediator is organizational dehumanization, which captures a negative psychological state, our model is more directly concerned with resource preservation, loss prevention, and the reduction of defensive resource-protection motives. When individuals face structural threats or actual resource loss, they may enter a loss-oriented process in which they become more motivated to conserve remaining resources and avoid further depletion. To cope with the resulting psychological strain, they activate the “resource defense principle, “adopting defensive, self-protective strategies to prevent further depletion. Building on this logic, the present study uses COR theory as the primary lens through which to explain how different forms of human–AI interaction shape employees’ subsequent behavioral responses.

Existing research has shown that AI exerts a double-edged effect in the workplace. On the one hand, AI can enhance work engagement and contribute to favorable work outcomes; on the other hand, it may also heighten employees’ job insecurity and perceived threat (Koo et al., 2021; Mitchell et al., 2019; Wang et al., 2023). In this regard, human–AI interaction is closely tied to employees’ experiences of resource preservation and resource depletion (Koo et al., 2021; Schmitz, 2013; Siemsen et al., 2008). Employees who perceive enhanced human–AI interaction may be more willing to invest resources in exchange for more valuable returns, whereas those who perceive impeded human–AI interaction may become more inclined to conserve their existing resources by reducing further investment. As employees collaborate with AI, they may thus experience either resource preservation or resource depletion, which in turn shapes their perceptions of organizational dehumanization and ultimately influences their work behaviors, including knowledge territorial behavior. COR theory is therefore well suited to explaining the theoretical model proposed in this study.

### 3.2 Human–AI interaction and knowledge territorial behavior

According to conservation of resources (COR) theory, individuals respond to changes in workplace resources by adopting behavioral strategies aimed either at preserving valued resources or at preventing further loss (Hobfoll et al., 2018). In digitally mediated work settings, different forms of human–AI interaction may trigger diverging behavioral strategies, thereby exerting asymmetric effects on employees’ knowledge territorial behavior.

Enhanced human–AI interaction is expected to reduce knowledge territorial behavior. Rather than arguing that enhanced human–AI interaction necessarily produces a full resource gain spiral, we draw on COR theory’s resource preservation and loss-prevention logic to suggest that enhanced interaction helps employees maintain and protect valued psychological resources. Under this pattern, AI relieves employees of routine tasks and supports their job autonomy, proactivity, and decision-making effectiveness (Tang et al., 2022; Wang et al., 2023). Because this interaction pattern reduces unnecessary resource depletion and helps employees complete tasks more effectively, employees are less likely to perceive AI as a threat to their status or irreplaceability. These work conditions protect employees’ sense of control, competence, and professional value, thereby reducing the likelihood that they will experience AI as a threat to their resources or irreplaceability. Because employees are less likely to experience resource loss or status devaluation, their motivation to activate defensive resource-protection strategies becomes weaker. Consequently, the inherent drive to activate resource defense mechanisms diminishes (Brown et al., 2014a,b). As a result, employees have less need to assert ownership through frequent knowledge marking or to restrict others’ access through knowledge defense, thereby reducing their overall tendency to engage in knowledge territorial behavior.

Accordingly, we propose the following hypothesis:

*H1a*: Enhanced human–AI interaction is negatively related to employees’ knowledge territorial behavior.

By contrast, impeded human–AI interaction is expected to increase knowledge territorial behavior. Impeded human–AI interaction reflects employees’ perception that AI obstructs task accomplishment, disrupts workflow, or increases the psychological and cognitive costs of work. Although such experiences often occur when algorithms dominate task allocation, monitoring, or decision-making, the critical issue is that AI involvement undermines employees’ autonomy, effectiveness, and resource states. This technology-related obstruction may lead to the erosion of cognitive and decision authority, the fragmentation and mechanization of job tasks, and a diminished sense of irreplaceability (Koo et al., 2021; Rinta-Kahila et al., 2023). When AI is experienced as inefficient, intrusive, or misaligned with work needs, employees may perceive stronger resource loss and become more motivated to protect the remaining high-value resources they still possess, namely, their unique knowledge. In an effort to establish clear resource boundaries under technological encroachment, employees may not only engage more strongly in knowledge marking to assert their irreplaceability, but may also adopt more aggressive forms of knowledge defense as a protective strategy to preserve their remaining value (Peng, 2013; Spence et al., 2014).

Accordingly, we propose the following hypothesis:

*H1b*: Impeded human–AI interaction is positively related to employees’ knowledge territorial behavior.

### 3.3 The mediating role of organizational dehumanization

Following our established theoretical framework (Hobfoll, 1989; Hobfoll et al., 2018), we conceptualize organizational dehumanization as a profound experience of resource depletion arising from the loss of core identity-related and psychological resources (Bell and Khoury, 2011). In the present study, we argue that enhanced and impeded human–AI interaction reshape employees’ work resources in fundamentally different ways, thereby eliciting distinct levels of organizational dehumanization and, in turn, different forms of knowledge territorial behavior.

Organizational dehumanization is expected to mediate the relationship between enhanced human–AI interaction and knowledge territorial behavior. Enhanced human–AI interaction emphasizes the supportive and augmenting role of technology. Under this pattern, employees retain control over core decisions and are not only relieved from the burden of routine tasks, but are also able to improve their work effectiveness by leveraging algorithmic capabilities (Huang and Gursoy, 2024; Tang et al., 2022; Wang et al., 2023). In line with the revised COR logic, we conceptualize this process primarily as the prevention of resource loss rather than as direct evidence of a resource gain spiral. Enhanced human–AI interaction reduces unnecessary resource expenditure and helps preserve employees’ autonomy, professional dignity, and sense of subjective agency. Such a resource-preserving environment reinforces employees’ subjective standing in the workplace and signals that the organization values their human intelligence and professional contribution. As a result, employees are less likely to experience themselves as objectified or treated merely as instruments for organizational ends (Bell and Khoury, 2011). By reducing organizational dehumanization, enhanced human–AI interaction mitigates a negative psychological state that would otherwise activate defensive resource-protection motives. Under such conditions, employees no longer need to treat tacit knowledge as a defensive asset for preserving their status, and their tendency to engage in knowledge territorial behavior is therefore reduced.

Accordingly, we propose the following hypothesis:

*H2a*: Organizational dehumanization mediates the relationship between enhanced human–AI interaction and employees’ knowledge territorial behavior, such that enhanced human–AI interaction reduces knowledge territorial behavior by decreasing organizational dehumanization.

Organizational dehumanization is also expected to mediate the relationship between impeded human–AI interaction and knowledge territorial behavior. In contrast to enhanced interaction, impeded human–AI interaction fundamentally undermines employees’ core psychological resources (Hobfoll, 1989; Hobfoll et al., 2018). When algorithmic systems take over core judgment and decision-making functions, employees are relegated to the role of technical subordinates who are continuously monitored, and critical resources such as work autonomy and skill variety are severely undermined. Although the immediate source of pressure appears to be technological, employees are likely to experience this pressure as organizationally meaningful because the AI system is introduced and legitimized by the organization. In other words, algorithmic control is not merely a technical feature; it represents an organizationally authorized work arrangement through which the organization allocates authority, defines employees’ roles, and determines how their labor should be evaluated and used (Jiang et al., 2026; Chen et al., 2026; Zoppelletto et al., 2026; Li et al., 2026). This persistent depletion of valued resources directly thrusts employees into a severe state of organizational dehumanization, whereby they perceive their human uniqueness and professional dignity as being erased and themselves as reduced to mere components of system operation (Andrighetto et al., 2017; Belmi and Schroeder, 2021). When employees find that their judgment is repeatedly overridden by AI, their work rhythm is dictated by algorithmic outputs, and their role is narrowed to executing system-generated instructions, they may infer that the organization values them less as autonomous professionals and more as instruments for efficiency and control. Therefore, the transition from algorithmic stress to organizational dehumanization occurs through employees’ attribution of AI-mediated control to the organization that deploys and authorizes the system. Because this dehumanized state embodies the ultimate form of psychological exhaustion, it serves as a powerful catalyst for the resource defense principle (Halbesleben et al., 2014; Hobfoll et al., 2018). In an effort to regain at least a minimal sense of control and self-worth in an algorithm-dominated environment, they may cling tightly to the limited high-value resources still under their control and engage in knowledge territorial behavior, such as withholding key information and constructing professional barriers, to resist complete marginalization by technology (Ahmed and Khalid Khan, 2016; Sarwar et al., 2021). Moreover, organizational dehumanization has been shown to foster negative interpersonal responses and to increase tendencies toward withdrawal and avoidance (Demoulin et al., 2021). Because dehumanization signals continued resource loss, employees may disengage from work relationships and adopt avoidance-based protective strategies to shield their remaining resources from further erosion (Ahmed and Khalid Khan, 2016; Bell and Khoury, 2011; Brison et al., 2024; Caesens and Brison, 2023). In this context, knowledge territorial behavior becomes more likely.

Accordingly, we propose the following hypothesis:

*H2b*: Organizational dehumanization mediates the relationship between impeded human–AI interaction and employees’ knowledge territorial behavior, such that impeded human–AI interaction increases knowledge territorial behavior by increasing organizational dehumanization.

### 3.4 The moderating role of paradoxical leadership

Drawing on conservation of resources (COR) theory and fairness heuristic theory, individuals’ psychological experiences and behavioral responses to resource fluctuations and uncertainty at work are deeply embedded in the broader pool of contextual resources provided by the organization. As a central provider of such contextual resources, leaders play a critical role in shaping how employees acquire, preserve, or lose valued resources. Paradoxical leadership integrates seemingly contradictory yet interdependent behavioral demands across multiple dimensions, including uniformity and individualization, self-centeredness and other-centeredness, control and autonomy, standardization and flexibility, as well as distance and closeness (Zhang, 2015). By accommodating these competing demands simultaneously, paradoxical leadership functions as a vital contextual resource pool that supplements employees’ psychological capital, enhances fairness perceptions, and helps employees navigate uncertainty in complex work settings. Accordingly, paradoxical leadership is likely to serve as an important boundary condition in the relationship between human–AI interaction and organizational dehumanization.

Paradoxical leadership is expected to strengthen the negative relationship between enhanced human–AI interaction and organizational dehumanization. Rather than operating in a vacuum, the resource-preserving effect of enhanced interaction is significantly amplified when supported by a rich contextual resource pool. Enhanced human–AI interaction helps employees preserve valued resources by reducing unnecessary task burdens and supporting autonomy and decision-making effectiveness (Hobfoll et al., 2018; Li et al., 2025). High levels of paradoxical leadership may further amplify this resource-preserving process through the flexible integration of control and autonomy as well as consistency and individualization. In such an integrative context, leaders not only inject additional psychological resources, such as self-efficacy and confidence, but also reduce employees’ fears of losing core resources (Avolio et al., 2004). At the same time, fairness heuristic theory suggests that the transparency and fairness conveyed by paradoxical leadership enable employees to interpret technologically enabled role changes in a more positive manner (Lind and Van den Bos, 2002). By providing both structural empowerment and interactional fairness, paradoxical leadership effectively signals that employees are highly valued, thereby magnifying the protective effect of enhanced interaction against feelings of being instrumentalized (Zhang, 2015; Niu et al., 2022). As a result, the suppressing effect of enhanced human–AI interaction on organizational dehumanization should become stronger under high levels of paradoxical leadership. By contrast, when paradoxical leadership is weak, leaders are less able to provide flexible contextual support, thereby reducing the extent to which enhanced interaction alleviates organizational dehumanization.

Accordingly, we propose the following hypothesis:

*H3a*: Paradoxical leadership moderates the relationship between enhanced human–AI interaction and organizational dehumanization, such that the negative relationship is stronger when paradoxical leadership is high rather than low.

Paradoxical leadership is also expected to weaken the positive relationship between impeded human–AI interaction and organizational dehumanization. Impeded human–AI interaction, characterized by algorithmic domination and intensive monitoring, undermines employees’ sense of agency and induces strong feelings of dehumanization and resource exhaustion (Li et al., 2025). We acknowledge that this buffering effect is not self-evident. Because paradoxical leadership itself involves the simultaneous expression of seemingly contradictory demands, such as control and autonomy, it could theoretically intensify role ambiguity and cognitive overload if employees perceive it as an additional layer of inconsistent control imposed on top of an already restrictive AI system. However, high levels of paradoxical leadership differ from contradictory or inconsistent supervision. Rather than simply adding more control, paradoxical leaders seek to reconcile competing demands by clarifying role expectations, explaining why efficiency-oriented systems are used, and preserving employees’ discretion and dignity within standardized work arrangements (Zhang, 2015; Zhang et al., 2015). Faced with such structural resource loss, high levels of paradoxical leadership may serve a critical buffering function in at least two ways. First, by balancing standardization and flexibility as well as distance and closeness, paradoxical leadership introduces a sense of humanized concern into otherwise impersonal algorithmic control (Sparr et al., 2022; Vaughn, 2017). Such leadership can reduce employees’ uncertainty by helping them understand the boundary between algorithmic recommendations and human judgment, thereby preventing AI-based control from being interpreted as complete organizational disregard for human agency. This concern can enhance employees’ psychological capital and alleviate the fear of resource loss caused by technological domination, which is a key antecedent of defensive behaviors such as knowledge withholding and territoriality (Brown et al., 2014a,b; Peng, 2013). Second, under conditions of algorithm-induced uncertainty and objectification, the interactional fairness provided by paradoxical leadership offers employees an important heuristic cue for interpreting their treatment within the organization (Zhang, 2015). When leaders transparently explain system requirements while simultaneously recognizing employees’ individual needs and professional judgment, employees are more likely to perceive that organizational control is accompanied by human concern rather than by pure instrumentalization. When employees perceive that leaders can transparently manage the tension between system control and human concern, they are more likely to believe that they continue to be regarded as valued organizational members rather than as mere data-processing tools, thereby weakening organizational dehumanization (Bell and Khoury, 2011). Moreover, high levels of paradoxical leadership may encourage knowledge exchange among team members and foster knowledge creation (Lee et al., 2010), transforming workplace knowledge from a private defensive resource into a shared collective asset and thereby undermining the defensive motives triggered by impeded interaction (Srivastava et al., 2006). In contrast, when paradoxical leadership is low, leaders may either passively defer to algorithmic control or impose rigid demands without providing explanation, flexibility, or individualized support. Under such conditions, impeded human–AI interaction is more likely to be interpreted as organizational objectification. Thus, the proposed buffering effect depends on paradoxical leadership functioning as integrative sensemaking and resource support, rather than as additional contradictory control.

Accordingly, we propose the following hypothesis:

*H3b*: Paradoxical leadership moderates the relationship between impeded human–AI interaction and organizational dehumanization, such that the positive relationship is weaker when paradoxical leadership is high rather than low.

Furthermore, because paradoxical leadership serves as a crucial contextual resource that shapes the initial impact of human–AI interaction on organizational dehumanization, it logically extends to condition the overall indirect effects on employees’ knowledge territorial behavior. Specifically, for enhanced human–AI interaction, high levels of paradoxical leadership strengthen the resource-preserving effect of AI support, thereby generating a stronger reduction in organizational dehumanization, which in turn mitigates the need for employees to activate the resource defense principle through knowledge territoriality (Smith and Lewis, 2011). Conversely, for impeded human–AI interaction, high levels of paradoxical leadership buffer the profound resource depletion (Zhang, 2015), preventing employees from falling into severe organizational dehumanization and subsequently reducing their self-protective tendency to withhold key information. Therefore, the indirect negative effect of enhanced human–AI interaction on knowledge territorial behavior through organizational dehumanization should be stronger when paradoxical leadership is high rather than low.

Accordingly, we propose the following hypothesis:

*H4a*: Paradoxical leadership moderates the indirect effect of enhanced human–AI interaction on knowledge territorial behavior through organizational dehumanization, such that the indirect negative effect is stronger when paradoxical leadership is high rather than low.

*H4b*: Paradoxical leadership moderates the indirect effect of impeded human–AI interaction on knowledge territorial behavior through organizational dehumanization, such that the indirect positive effect is weaker when paradoxical leadership is high rather than low.

**Figure 1:**
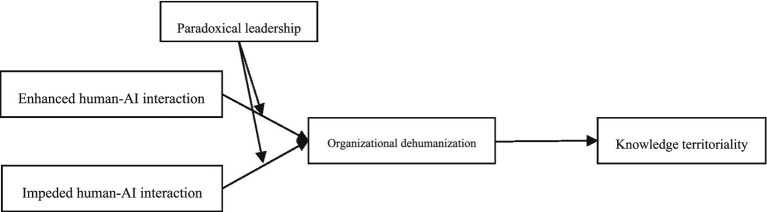
Research model.

The proposed research model is presented in Figure 1.

Although the theoretical rationale underlying our hypotheses is clear, relying solely on cross-sectional survey methods precludes strict causal inferences and leaves the findings vulnerable to unobserved confounders and reverse causality. To address these methodological limitations, we employed a multi-study design comprising a scenario-based experiment (Study 1) and a two-wave field survey (Study 2) to systematically test our theoretical model. Specifically, Study 1 utilized an experimental design to manipulate the forms of human–AI interaction. By randomly assigning participants and tightly controlling extraneous variables, this study facilitated clean causal inferences and established the causal direction of our core hypotheses. Subsequently, Study 2 was conducted in field settings to test the complete moderated mediation model—including the boundary role of paradoxical leadership—within real organizational contexts, thereby enhancing the external and ecological validity of our findings. Overall, these two studies are highly complementary: Study 1 maximizes internal validity and causal identification, whereas Study 2 establishes external validity and captures the complexity of the full model. Together, this dual-method approach provides rigorous and robust empirical support for our propositions.

## 4 Study 1: scenario-based experiment

### 4.1 Method

#### 4.1.1 Participants and design procedure

Study 1 employed a scenario-based experimental design to rigorously control for extraneous variables and to establish the causal effects of enhanced and impeded human-AI interaction on employees’ organizational dehumanization and knowledge territorial behavior, thereby addressing the limitations of traditional survey research in causal inference.

The target population of this study consisted of employees working in Chinese high-technology firms that had adopted human–AI collaborative work practices. Through alumni networks, the researchers contacted several large internet companies and obtained their cooperation after clearly explaining the purpose of the study and the anonymization procedures. The inclusion of these firms enhanced the ecological validity of the experiment for two reasons. First, these firms were highly similar in terms of business nature, organizational scale, and industry background, and all had extensively integrated AI into their work processes, meaning that employees generally had genuine experience with human–AI collaboration. Second, both enhanced and impeded forms of human–AI interaction objectively coexisted in their actual work settings, which made it easier for participants to immerse themselves in the experimental scenarios and thus strengthened the effectiveness of the manipulation.

An *a priori* power analysis was conducted using G*Power 3.1 for a one-factor, three-group between-subjects design. With the significance level set at *α* = 0.05, statistical power at 1 − *β* = 0.80, and a medium effect size specified as *f* = 0.25 following Cohen (1988), the results indicated that a minimum total sample size of 159 participants was required to detect mean differences across the three groups, corresponding to approximately 53 participants per group. Considering the possibility of invalid responses in scenario-based experiments, the study ultimately planned to recruit 90 participants for each group.

A total of 256 valid responses were obtained in the final experiment. Among the valid participants, 51.953% were male and 48.047% were female. In terms of age, 18.750% were aged 26–30 years, 44.141% were aged 31–35 years, 25.391% were aged 36–40 years, and 11.719% were aged 41 years or older. Regarding education, 30.469% had a junior college degree or below, 44.531% held a bachelor’s degree, 17.188% held a master’s degree, and 7.813% held a doctoral degree. With respect to organizational tenure, 5.078% had worked for less than 1 year, 10.547% for 1–3 years, 25.391% for 3–5 years, 38.281% for 5–10 years, and 20.703% for more than 10 years. Participants were randomly assigned by the system to one of three conditions: the enhanced human-AI interaction group (*N* = 84), the neutral human–AI interaction group (*N* = 85), and the impeded human-AI interaction group (*N* = 87).

#### 4.1.2 Experimental design and procedure

Study 1 adopted a one-factor, three-level scenario-based experimental design in which human–AI interaction was manipulated across three conditions: enhanced, neutral, and impeded. To assess the effectiveness of the manipulation, all participants were asked to complete the enhanced human-AI interaction scale and the impeded human-AI interaction scale after reading the scenario materials. It was expected that participants in the enhanced condition would report significantly higher levels of enhanced interaction perception than those in the neutral and impeded conditions, whereas participants in the impeded condition would report significantly higher levels of impeded interaction perception than those in the neutral and enhanced conditions. The neutral condition was expected to fall between the other two conditions on both dimensions. Differences across the three groups on the outcome variables were then compared to test the effects of different forms of human–AI interaction.

The experimental scenarios were adapted from the core features of the two-dimensional scale developed and validated by Li et al. (2025). The researchers generated an online experiment link using the Wenjuanxing platform and distributed it to employees in the participating firms described above.

Participants were first presented with the following background scenario: they were asked to imagine that they were employees in the operations department of a large internet company, primarily responsible for the day-to-day operation and management of a particular business area. Their regular work involved collecting and organizing business data, tracking task progress, analyzing problems, developing solutions, and making decisions and executing tasks based on specific circumstances.

To improve work efficiency, the company had recently introduced an AI system to assist employees in their daily work. This AI system could access relevant information from the business platform, process data, and participate in task execution procedures. Participants were told that they had already been using this AI system in their work for some time and had gradually become accustomed to this form of human–AI collaboration. They were then instructed to read one of the following scenarios and imagine themselves in that work situation.

In the enhanced human-AI interaction condition, the AI system was described as primarily undertaking data acquisition, information collection, and basic data organization. Many tasks that previously required substantial time and effort, such as data retrieval, information summarization, and spreadsheet compilation, could now be completed automatically by the AI system. In addition, the system assisted with tedious and repetitive tasks, such as categorizing, organizing, and conducting preliminary screening of routine information, thereby helping employees move more quickly to subsequent work stages. The AI system clearly reduced the time participants needed to spend on mechanical tasks, allowing them to devote more attention to more important activities such as problem analysis, judgment, and work planning. Under this work arrangement, the AI system was framed primarily as a source of support and assistance that reduced workload and enhanced efficiency, while key judgments and major work advancement remained under the employee’s control.

In the neutral human–AI interaction condition, the AI system was described as participating in some data processing and information support functions, such as routine information retrieval, basic data organization, and task progress display. For certain routine tasks, the AI system provided a degree of technical support, but its role was mainly limited to conventional assistance. In most cases, participants still needed to rely on their own experience to conduct further analysis, make judgments, and complete subsequent processing based on the information provided by the AI system. Although the system participated in the workflow, it neither substantially reduced workload nor directly dominated the handling or decision-making of important tasks. Overall, the AI system was portrayed as a regular tool embedded in daily work that neither clearly enhanced employees’ autonomy and ease nor noticeably interfered with their normal work.

In the impeded human-AI interaction condition, the AI system was described as not only participating in data processing but also autonomously making judgments from among multiple alternative options and directly determining how certain tasks should be handled. For some important tasks, the AI system provided decisions in advance, while the employee’s role was primarily to execute and cooperate with those decisions. As the role of the AI system became increasingly prominent in the workflow, participants were expected to advance their work according to the system’s judgments, even when they believed that other approaches might be more appropriate. Because the AI system intervened in key task decisions, participants were likely to feel that their normal work rhythm was disrupted. Under this work arrangement, the AI system not only failed to reduce workload, but instead increased pressure. Participants were required to continually adapt to the system’s decisions and work arrangements, making this form of human–AI collaboration a source of additional tension and burden.

After reading the scenario materials, participants first completed manipulation-check items to confirm that the scenarios had successfully elicited the intended perceptions. They were then instructed to answer the measures of organizational dehumanization and knowledge territorial behavior based on how they would genuinely feel in the described situation. Finally, they completed the demographic questionnaire.

#### 4.1.3 Measures

To ensure linguistic equivalence, the original English scales were translated into Chinese using the translation–back-translation procedure. The translated questionnaire was then reviewed by one professor and two doctoral researchers in the fields of organizational behavior and human resource management, after which minor revisions were made before formal administration.

*Human–AI interaction*: Human–AI interaction was measured using the two-dimensional scale developed and validated by Li et al. (2025). Four items were used to assess enhanced human-AI interaction, with a representative item being, “In my daily work, artificial intelligence is primarily responsible for autonomous data acquisition and collection.” Four additional items were used to assess impeded human-AI interaction, with a representative item being, “In my daily work, artificial intelligence is primarily responsible for autonomously making decisions from alternative options.” All items were rated on a five-point Likert scale. Cronbach’s alpha was 0.842 for the enhanced human-AI interaction scale and 0.874 for the impeded human-AI interaction scale.

*Organizational dehumanization*: Organizational dehumanization was measured using the five-item scale adapted by Lagios et al. (2024) from Caesens et al. (2017). A representative item is, “My organization treats me as a tool to achieve its goals.” All items were rated on a five-point Likert scale. The scale demonstrated good internal consistency, with a Cronbach’s alpha of 0.893.

Knowledge territorial behavior. Knowledge territorial behavior was measured using a four-item scale adapted from Avey et al.’s (2009) territoriality measure and subsequently applied to the knowledge territorial behavior context by Song and Cao (2015). A representative item is, “I feel that I need to protect my knowledge from being used by others in my organization.” All items were rated on a five-point Likert scale. The scale showed good internal consistency, with a Cronbach’s alpha of 0.879.

Control variables. Based on prior research suggesting that gender, age, education level, and organizational tenure may influence territorial behavior and knowledge hiding (Singh, 2019; Yang et al., 2024), these variables were included as controls in the analyses.

### 4.2 Results

#### 4.2.1 Common method Bias

Although the present study adopted a scenario-based experimental design, in which random assignment and scenario manipulation helped reduce the potential influence of common method bias to some extent, the major variables were still derived from participants’ self-reports collected at the same point in time. It was therefore necessary to assess the extent of common method bias. Following the recommendations of Podsakoff et al. (2003), we employed both Harman’s single-factor test and a single-factor confirmatory factor analysis to examine this issue.

A single-factor measurement model was estimated in the confirmatory factor analysis. The results showed that the fit of the single-factor model was poor, with χ^2^/df = 7.676, CFI = 0.677, TLI = 0.631, RMSEA = 0.162, and SRMR = 0.111, all of which were well below acceptable thresholds. These findings indicate that the measurement items did not adequately load onto a single latent construct. Taken together, the results suggest that common method bias was unlikely to pose a serious concern in the present study.

#### 4.2.2 Descriptive statistics and correlation analysis

Table 1 reports the means, standard deviations, and correlation coefficients for all study variables. The results indicate that enhanced human-AI interaction was significantly negatively correlated with organizational dehumanization (*r* = −0.517, *p* < 0.001) and knowledge territorial behavior (*r* = −0.487, *p* < 0.001). Impeded human-AI interaction was significantly positively correlated with organizational dehumanization (*r* = 0.443, *p* < 0.001) and knowledge territorial behavior (*r* = 0.461, *p* < 0.001). In addition, organizational dehumanization was significantly positively correlated with knowledge territorial behavior (*r* = 0.492, *p* < 0.001).

**Table 1:** Descriptive statistical analysis and correlation analysis.

Variables	Mean	SD	1	2	3	4
1. EHI	0.328	0.470	1			
2. IHI	0.340	0.475	−0.501***	1		
3. OD	3.487	1.174	−0.517***	0.461***	1	
4. KT	3.569	1.239	−0.487***	0.443***	0.492***	1

EHI, enhanced human-AI interaction; IHI, impeded human-AI interaction; OD, organizational dehumanization; KT, knowledge territorial behavior; SD, standard deviation. *p* < 0.05, *p* < 0.01, *p* < 0.001. The enhanced human-AI interaction group was coded as 1 and all other groups as 0; the impeded human-AI interaction group was coded as 1 and all other groups as 0.

Taken together, these findings provide initial support for the proposed hypotheses. Specifically, enhanced human-AI interaction may reduce employees’ knowledge territorial behavior by lowering organizational dehumanization, whereas impeded human-AI interaction may increase employees’ knowledge territorial behavior by heightening organizational dehumanization. These results offer a preliminary basis for the subsequent hypothesis testing.

#### 4.2.3 Confirmatory factor analysis

To examine the discriminant validity of the core constructs, we conducted confirmatory factor analyses in AMOS 29.0 for enhanced human-AI interaction, impeded human-AI interaction, organizational dehumanization, and knowledge territorial behavior. The hypothesized four-factor model was then compared with several alternative models in which some constructs were combined.

As shown in Table 2, the four-factor model demonstrated good fit to the data, with χ^2^ = 148.074, df = 113, χ^2^/df = 1.310, CFI = 0.986, TLI = 0.983, RMSEA = 0.035, and SRMR = 0.037. By contrast, the three-factor, two-factor, and one-factor models, in which some constructs were merged, all exhibited substantially poorer fit. These results indicate that enhanced human-AI interaction, impeded human-AI interaction, organizational dehumanization, and knowledge territorial behavior are empirically distinct constructs, thereby providing support for discriminant validity and justifying the subsequent analyses.

**Table 2:** The result of confirmatory factor analysis.

Model	Factor	χ^2^	df	χ^2^/df	RMSEA	CFI	TLI	SRMR
Basic model	EHI, IHI, OD, KT	148.074	113	1.310	0.035	0.986	0.983	0.037
Three-factor model	EHI + IHI, OD, KT	328.628	116	2.833	0.085	0.914	0.899	0.065
Bifactor model	EHI + IHI + OD, KT	584.588	118	4.954	0.125	0.811	0.782	0.083
One-factor model	All variables loaded onto a single factor.	913.491	119	7.676	0.162	0.677	0.631	0.111

EHI, enhanced human-AI interaction; IHI, impeded human-AI interaction; OD, organizational dehumanization; KT, knowledge territorial behavior.

#### 4.2.4 Hypothesis testing

##### 4.2.4.1 Manipulation checks

To assess the effectiveness of the scenario manipulation, we first compared the three experimental groups in terms of perceived enhanced human-AI interaction and perceived impeded human-AI interaction. The results of one-way ANOVA showed that the three groups differed significantly in perceived enhanced human-AI interaction, *F* = 117.572, *p* < 0.001. *Post hoc* comparisons further indicated that participants in the enhanced condition reported significantly higher levels of enhanced interaction perception than those in the neutral and impeded conditions. The three groups also differed significantly in perceived impeded human-AI interaction, *F* = 71.023, *p* < 0.001. *Post hoc* comparisons showed that participants in the hindering condition reported significantly higher levels of impeded interaction perception than those in the neutral and enhanced conditions. These findings suggest that the scenario manipulation was successful.

##### 4.2.4.2 Main effects

Because Study 1 employed a one-factor, three-level experimental design, two dummy variables were created using the neutral group as the reference category: the enhanced human-AI interaction group was coded as 1 and all other groups as 0, and the impeded human-AI interaction group was coded as 1 and all other groups as 0.

After controlling for gender, age, education level, and organizational tenure, regression analyses were conducted with knowledge territorial behavior as the dependent variable. As shown in Table 3, the enhanced human-AI interaction group had a significant negative effect on employees’ knowledge territorial behavior (*β* = −0.480, *p* < 0.001), indicating that, relative to the neutral condition, the enhanced human-AI interaction scenario significantly reduced employees’ knowledge territorial behavior. Thus, H1a was supported. In contrast, the impeded human-AI interaction group had a significant positive effect on employees’ knowledge territorial behavior (*β* = 0.426, *p* < 0.001), indicating that, relative to the neutral condition, the impeded human-AI interaction scenario significantly increased employees’ knowledge territorial behavior. Thus, H1b was supported.

**Table 3:** Hierarchical regression results.

Variables	KT				OD		
	M1	M2	M3	M4	M5	M6	M7
Gender	0.027	−0.040	−0.018	−0.021	0.098	0.027	0.052
Age	0.163**	0.110*	0.108	0.150**	0.027	−0.029	−0.031
Education level	−0.017	0.030	0.010	0.012	−0.060	−0.011	−0.032
Organizational tenure	−0.108	−0.026	−0.058	−0.040	−0.139*	−0.053	−0.087
EHI		−0.480***				−0.506***	
IHI			0.426***				0.447***
OD				0.487***			
R^2^	0.038	0.253	0.212	0.267	0.033	0.272	0.225
ΔR^2^	0.038	0.215	0.174	0.229	0.033	0.239	0.192
F	2.450	16.893	13.423	18.181	2.167	18.719	14.510

EHI, enhanced human-AI interaction; IHI, impeded human-AI interaction; OD, organizational dehumanization; KT, knowledge territorial behavior. *p* < 0.05, *p* < 0.01, *p* < 0.001.

##### 4.2.4.3 Mediation effects

To test the mediating role of organizational dehumanization, we used Hayes’s (2018) PROCESS macro Model 4 with 5,000 bootstrap resamples. Using the neutral group as the reference category, Table 4 reports the indirect effects of the enhanced human-AI interaction group and the impeded human-AI interaction group on knowledge territorial behavior through organizational dehumanization.

**Table 4:** Results of the direct and indirect effects.

Hypothesized Path	Effect	BootSE	95% BootCI
EHI → OD → KT	−0.447	0.099	[−0.656, −0.266]
IHI → OD → KT	0.434	0.094	[0.269, 0.638]

As shown in Table 3, the enhanced human-AI interaction group had a significant negative effect on organizational dehumanization (*β* = −0.506, *p* < 0.001), whereas organizational dehumanization had a significant positive effect on knowledge territorial behavior (*β* = 0.487, *p* < 0.001). Further bootstrap analyses indicated that the indirect effect of enhanced human-AI interaction on knowledge territorial behavior through organizational dehumanization was −0.447, with a 95% confidence interval of [−0.656, −0.266], excluding zero. This result suggests that organizational dehumanization significantly mediated the relationship between enhanced human-AI interaction and knowledge territorial behavior. In other words, enhanced human-AI interaction reduced employees’ knowledge territorial behavior by lowering their organizational dehumanization. Thus, H2a was supported.

At the same time, the impeded human-AI interaction group had a significant positive effect on organizational dehumanization (*β* = 0.447, *p* < 0.001). The bootstrap results further showed that the indirect effect of impeded human-AI interaction on knowledge territorial behavior through organizational dehumanization was 0.434, with a 95% confidence interval of [0.269, 0.638], which did not include zero. This finding indicates that organizational dehumanization significantly mediated the relationship between impeded human-AI interaction and knowledge territorial behavior. That is, impeded human-AI interaction increased employees’ knowledge territorial behavior by heightening their organizational dehumanization. Thus, H2b was supported.

Taken together, the results of Study 1 indicate that enhanced human-AI interaction significantly reduced employees’ knowledge territorial behavior, whereas impeded human-AI interaction significantly increased it. Moreover, organizational dehumanization played a significant mediating role in these relationships. Thus, H1a, H1b, H2a, and H2b were all supported.

## 5 Study 2: survey study

### 5.1 Method

#### 5.1.1 Participants and design procedure

As many Chinese firms have now introduced AI to collaborate with employees, this work arrangement falls squarely within the domain of human–AI interaction. Accordingly, the target population of Study 2 consisted of employees working in Chinese firms that had adopted human–AI collaborative practices. Through alumni networks, the researchers contacted high-technology firms engaged in new-materials research and development that had already implemented AI-assisted work systems. Employees in these firms had direct experience with human–AI interaction, and the participating firms were relatively homogeneous in terms of business nature, organizational scale, and industry characteristics. Because these firms operate in R&D-intensive settings where proprietary knowledge, intellectual property boundaries, and confidentiality requirements are particularly salient, we explicitly distinguished organization-mandated knowledge protection from employees’ discretionary knowledge territorial behavior when designing Study 2. Organization-mandated knowledge protection refers to employees’ compliance with formal confidentiality rules, intellectual property policies, or firm-level secrecy requirements. By contrast, discretionary knowledge territorial behavior refers to employees’ individual tendency to mark, claim, and defend knowledge boundaries in everyday intraorganizational interactions. This distinction helped ensure that the focal construct of Study 2 remained employees’ psychologically driven knowledge territorial behavior rather than formal IP protection behavior required by the organization.

To reduce the potential influence of common method bias, several procedural remedies were adopted. First, data were collected in two waves with a one-month interval. The first-wave survey was administered in February 2026 and measured demographic variables, enhanced human-AI interaction, impeded human-AI interaction, and paradoxical leadership. The second-wave survey was administered in March 2026 and measured organizational dehumanization and knowledge territorial behavior. To ensure accurate matching of the two surveys, respondents were asked to provide the last four digits of their mobile phone number. Second, the questionnaires were completed anonymously in order to reduce respondents’ concerns. Third, a pilot test was conducted prior to the formal survey to evaluate whether the questionnaire items were clearly and appropriately worded, and problematic items were revised accordingly.

The survey yielded the following responses. In the first wave, 625 questionnaires were collected, of which 600 were retained as valid after excluding responses with excessively short completion times or obvious response patterns. In the second wave, 558 questionnaires were collected, of which 540 were valid. After matching the two-wave responses, the final sample consisted of 529 valid cases, yielding an overall effective response rate of 84.64%. Among the valid respondents, 51.040% were male and 48.960% were female. In terms of age, 46.881% were aged 26–30 years, 26.465% were aged 31–35 years, 14.556% were aged 36–40 years, and 12.098% were aged 41 years or older. Regarding education, 26.843% had a junior college degree or below, 45.936% held a bachelor’s degree, 18.715% held a master’s degree, and 8.507% held a doctoral degree. With respect to organizational tenure, 7.750% had worked for less than 1 year, 14.556% for 1–3 years, 19.660% for 3–5 years, 28.922% for 5–10 years, and 29.112% for more than 10 years.

#### 5.1.2 Measures

The original English scales were translated into Chinese using the translation–back-translation procedure. The translated questionnaire was then reviewed by one professor and two doctoral researchers in organizational behavior and human resource management, and revised before formal administration.

*Human–AI interaction*: Human–AI interaction was measured using the two-dimensional scale developed and validated by Li et al. (2025). Four items measured enhanced human-AI interaction, with a representative item being, “In my daily work, artificial intelligence is primarily responsible for autonomous data acquisition and collection.” Four additional items measured impeded human-AI interaction, with a representative item being, “In my daily work, artificial intelligence is primarily responsible for autonomously making decisions from alternative options.” All items were rated on a five-point Likert scale. Cronbach’s alpha was 0.852 for the enhanced human-AI interaction scale and 0.892 for the impeded human-AI interaction scale.

*Organizational dehumanization*: Organizational dehumanization was measured using the five-item scale adapted by Lagios et al. (2024) from Caesens et al. (2017). A representative item is, “My organization treats me as a tool to achieve its goals.” All items were rated on a five-point Likert scale. The scale showed good internal consistency, with a Cronbach’s alpha of 0.898.

*Paradoxical leadership*: Paradoxical leadership was measured using the 22-item scale developed by Zhang et al. (2015). All items used “during the past week” as the temporal anchor. A representative item is, “My supervisor shows leadership authority, but allows others to share leadership roles.” All items were rated on a five-point Likert scale. The scale demonstrated high internal consistency, with a Cronbach’s alpha of 0.965.

*Knowledge territorial behavior*: Knowledge territorial behavior was measured using a four-item scale adapted from Avey et al.’s (2009) territoriality measure and subsequently applied to the knowledge territorial behavior context by Song and Cao (2015). A representative item is, “I feel that I need to protect my knowledge from being used by others in my organization.” All items were rated on a five-point Likert scale. The scale showed good internal consistency, with a Cronbach’s alpha of 0.863. Consistent with prior research on knowledge territoriality, these items capture employees’ subjective and discretionary tendency to protect knowledge they perceive as personally valuable within the organization. Importantly, the items do not ask respondents to report compliance with formal confidentiality rules, intellectual property regulations, or organization-mandated secrecy requirements. Thus, the measure captures individual-level knowledge territorial behavior rather than formal IP protection behavior.

*Control variables*: Based on prior research suggesting that gender, age, education level, and organizational tenure may influence territoriality and knowledge hiding (Singh, 2019; Yang et al., 2024), these variables were included as controls in the analyses.

### 5.2 Results

#### 5.2.1 Common method Bias

Because the data in Study 2 were primarily derived from employees’ self-reported questionnaires, both procedural and statistical remedies were adopted to reduce the potential influence of common method bias. At the procedural level, the researchers used anonymous measurement, emphasized that the survey was intended solely for academic purposes, dispersed the order of questionnaire items, and included attention-check items to minimize respondents’ social desirability bias and consistency motives. At the statistical level, Harman’s single-factor test was first conducted through exploratory factor analysis using all measurement items. The results showed that five factors had eigenvalues greater than 1, and the first unrotated factor accounted for 38.003% of the total variance, which is below the commonly used threshold of 40%, suggesting that common method bias was not severe. In addition, a single-factor confirmatory factor analysis model was estimated. The results showed that the fit of the single-factor model was poor, with χ^2^/df = 7.610, CFI = 0.650, TLI = 0.631, RMSEA = 0.112, and SRMR = 0.135, all of which failed to meet acceptable fit standards. Taken together, these findings indicate that common method bias was unlikely to pose a serious problem in the present study.

#### 5.2.2 Descriptive statistics and correlation analysis

Table 5 presents the means, standard deviations, and correlations among the main study variables. The results indicate that enhanced human-AI interaction was significantly negatively correlated with organizational dehumanization (*r* = −0.447, *p* < 0.001) and knowledge territorial behavior (*r* = −0.485, *p* < 0.001). Impeded human-AI interaction was significantly positively correlated with organizational dehumanization (*r* = 0.472, *p* < 0.001) and knowledge territorial behavior (*r* = 0.491, *p* < 0.001). Organizational dehumanization was also significantly positively correlated with knowledge territorial behavior (*r* = 0.521, *p* < 0.001). In addition, paradoxical leadership was significantly negatively correlated with organizational dehumanization (*r* = −0.326, *p* < 0.001) and knowledge territorial behavior (*r* = −0.314, *p* < 0.001). Moreover, the square roots of the average variance extracted (AVE) for all constructs exceeded the corresponding inter-construct correlations, providing preliminary support for discriminant validity.

**Table 5:** Descriptive statistical analysis and correlation analysis.

Variables	Mean	SD	1	2	3	4	5	6	7	8	9
1. Gender	1.490	0.500	1								
2. Age	3.435	0.883	−0.093*	1							
3. Education level	2.089	0.889	−0.038	0.033	1						
4. Organizational tenure	3.571	1.260	−0.054	0.721***	−0.416***	1					
5. EHI	3.192	1.196	−0.068	0.090*	−0.023	0.107*	1				
6. IHI	3.570	1.244	0.020	−0.010	−0.001	−0.063	−0.342***	1			
7. OD	3.742	1.157	−0.020	−0.032	−0.047	−0.042	−0.447***	0.472***	1		
8. KT	3.691	1.147	−0.038	−0.037	0.008	−0.082	−0.485***	0.491***	0.521***	1	
9. PL	3.236	1.088	0.014	0.035	−0.030	0.069	0.337***	−0.289***	−0.326***	−0.314***	1
CR	–	–	–	–	–	–	0.855	0.893	0.900	0.865	0.965
AVE	–	–	–	–	–	–	0.597	0.677	0.642	0.616	0.559

EHI, enhanced human-AI interaction; IHI, impeded human-AI interaction; OD, organizational dehumanization; KT, knowledge territorial behavior; PL, paradoxical leadership; SD, standard deviation. *p* < 0.05, *p* < 0.01, *p* < 0.001. CR, composite reliability; AVE, average variance extracted.

#### 5.2.3 Confirmatory factor analysis

To examine the discriminant validity among the five core constructs, we conducted confirmatory factor analyses in AMOS 29.0 for enhanced human-AI interaction, impeded human-AI interaction, organizational dehumanization, knowledge territorial behavior, and paradoxical leadership. The hypothesized five-factor model was then compared with several alternative models in which some constructs were combined.

As shown in Table 6, the five-factor model demonstrated good fit to the data, with χ^2^ = 942.656, df = 692, χ^2^/df = 1.362, CFI = 0.981, TLI = 0.934, RMSEA = 0.026, and SRMR = 0.027. In contrast, the four-factor, three-factor, two-factor, and one-factor models, in which some constructs were merged, all showed substantially poorer fit. These findings indicate that enhanced human-AI interaction, impeded human-AI interaction, organizational dehumanization, knowledge territorial behavior, and paradoxical leadership are empirically distinct constructs, thereby providing strong support for discriminant validity.

**Table 6:** The result of confirmatory factor analysis.

Model	Factor	χ^2^	df	χ^2^/df	RMSEA	CFI	TLI	SRMR
Basic model	EHI, IHI, OD, KT, PL	942.656	692	1.362	0.026	0.981	0.934	0.027
Four-factor model	EHI + IHI, OD, KT, PL	1772.401	696	2.547	0.054	0.919	0.914	0.064
Three-factor model	EHI + IHI + OD, KT, PL	2429.313	699	3.475	0.068	0.870	0.862	0.058
Bifactor model	EHI + IHI + OD + KT, PL	2836.076	701	4.046	0.076	0.839	0.830	0.059
One-factor model	All variables loaded onto a single factor.	5342.537	702	7.610	0.112	0.650	0.631	0.135

EHI, enhanced human-AI interaction; IHI, impeded human-AI interaction; OD, organizational dehumanization; KT, knowledge territorial behavior; PL, paradoxical leadership.

#### 5.2.4 Hypothesis testing

To test the main effects, mediating effects, moderating effects, and moderated mediation effects, we employed hierarchical regression analysis and bootstrap procedures. Prior to testing the interaction effects, enhanced human-AI interaction, impeded human-AI interaction, and paradoxical leadership were mean-centered to reduce potential multicollinearity. We then created two interaction terms: enhanced human-AI interaction × paradoxical leadership and impeded human-AI interaction × paradoxical leadership. All regression models controlled for gender, age, education level, and organizational tenure.

##### 5.2.4.1 Main effects

To test H1a and H1b, hierarchical regression analyses were conducted with knowledge territorial behavior as the dependent variable. As shown in Table 7, after controlling for demographic variables, enhanced human-AI interaction had a significant negative effect on knowledge territorial behavior (*β* = −0.486, *p* < 0.001), indicating that the more strongly employees perceived enhanced human-AI interaction, the lower their level of knowledge territorial behavior. Thus, H1a was supported. At the same time, impeded human-AI interaction had a significant positive effect on knowledge territorial behavior (*β* = 0.487, *p* < 0.001), indicating that the more strongly employees perceived impeded human-AI interaction, the higher their level of knowledge territorial behavior. Thus, H1b was supported.

**Table 7:** Hierarchical regression results.

Variables	KT				OD						
	M1	M2	M3	M4	M5	M6	M7	M8	M9	M10	M11
Gender	−0.042	−0.072	−0.051	−0.029	−0.025	−0.052	−0.034	−0.045	−0.038	−0.029	−0.033
Age	0.091	0.095	0.024	0.055	0.068	0.072	0.004	0.063	0.051	0.004	0.007
Education level	−0.071	−0.063	−0.029	−0.016	−0.107	−0.099	−0.066	−0.097	−0.097	−0.068	−0.076
Organizational tenure	−0.180^*^	−0.129	−0.083	−0.109	−0.137	−0.090	−0.044	−0.076	−0.066	−0.034	−0.047
EHI		−0.486^***^				−0.450^***^		−0.384^***^	−0.384^***^		
IHI			0.487^***^				0.470^***^		−0.212^***^	0.411^***^	0.412^***^
OD				0.517^***^							
PL								0.470^***^	−0.212^***^		−0.186^***^
EHI × PL									−0.139^***^		
IHI × PL										−0.207^***^	−0.184^***^
R^2^	0.012	0.245	0.247	0.277	0.009	0.208	0.227	0.242	0.261	0.267	0.300
ΔR^2^	0.012	0.233	0.235	0.265	0.009	0.199	0.218	0.034	0.019	0.039	0.033
F	1.650	33.946	34.316	40.147	1.199	27.538	30.804	27.845	26.338	31.637	31.904

EHI, enhanced human-AI interaction; IHI, impeded human-AI interaction; OD, organizational dehumanization; KT, knowledge territorial behavior; PL, paradoxical leadership. Standardized regression coefficients (β) are reported in the table, and all variables included in the interaction terms were mean-centered prior to analysis. *p* < 0.05, *p* < 0.01, *p* < 0.001.

##### 5.2.4.2 Mediation effects

To examine the mediating role of organizational dehumanization, we used Hayes’s (2018) PROCESS macro Model 4 with 5,000 bootstrap resamples.

As reported in Table 7, enhanced human-AI interaction had a significant negative effect on organizational dehumanization (*β* = −0.450, *p* < 0.001), whereas organizational dehumanization had a significant positive effect on knowledge territorial behavior (*β* = 0.517, *p* < 0.001). The bootstrap results further showed that the indirect effect of enhanced human-AI interaction on knowledge territorial behavior through organizational dehumanization was −0.162, with a 95% confidence interval of [−0.209, −0.117], which did not include zero. These findings indicate that organizational dehumanization significantly mediated the relationship between enhanced human-AI interaction and knowledge territorial behavior. Therefore, H2a was supported.

Similarly, impeded human-AI interaction had a significant positive effect on organizational dehumanization (*β* = 0.470, *p* < 0.001). The bootstrap results showed that the indirect effect of impeded human-AI interaction on knowledge territorial behavior through organizational dehumanization was 0.161, with a 95% confidence interval of [0.111, 0.209], which did not include zero. This result indicates that organizational dehumanization significantly mediated the relationship between impeded human-AI interaction and knowledge territorial behavior. Therefore, H2b was supported.

##### 5.2.4.3 Moderation effects

**Figure 2:**
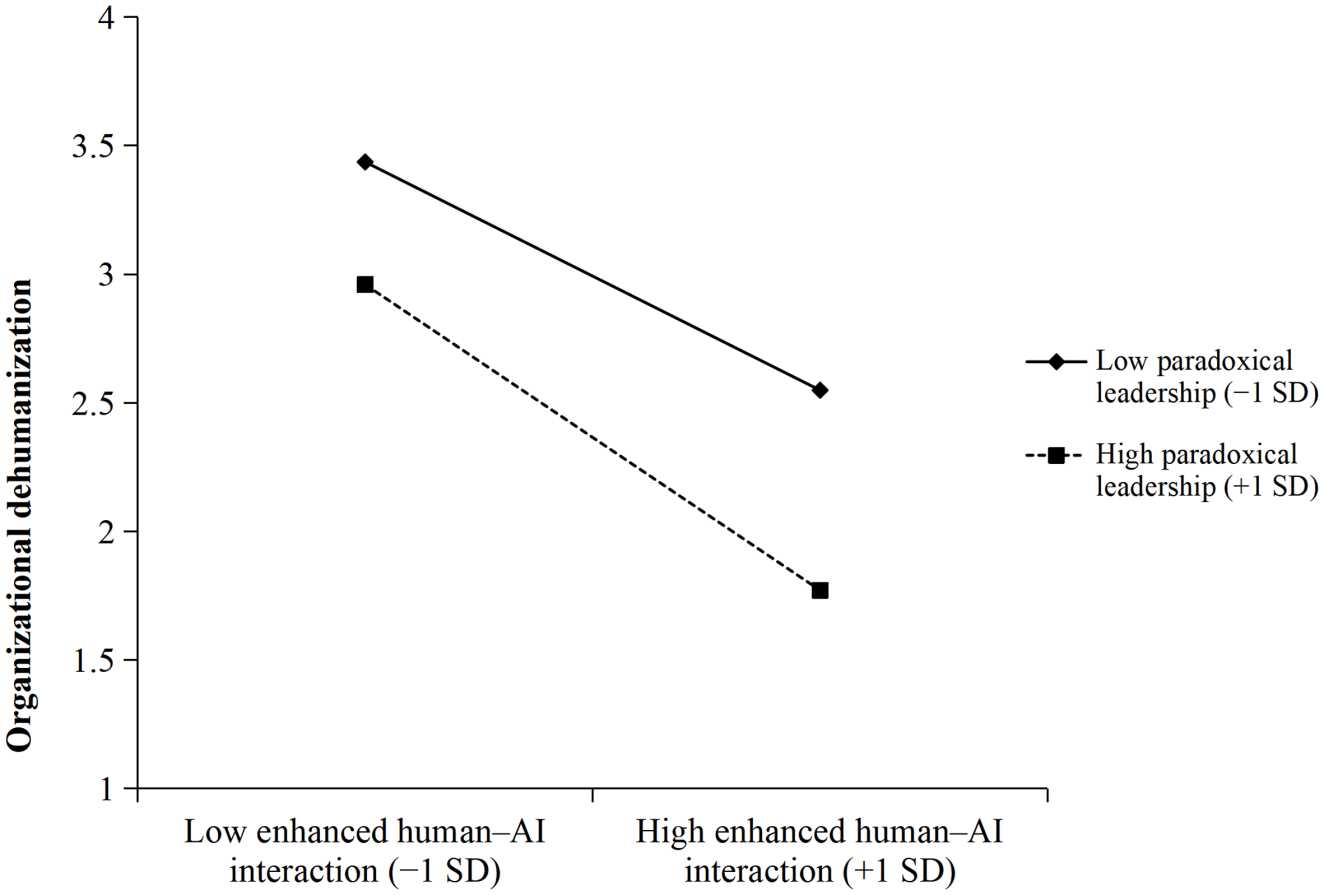
The moderating effect of paradoxical leadership on the relationship between enhanced human-AI interaction.

To examine the moderating role of paradoxical leadership, we employed hierarchical regression analyses with organizational dehumanization as the dependent variable. After controlling for the main effects, the interaction terms were entered sequentially into the regression models. As shown in Table 7, the interaction between enhanced human-AI interaction and paradoxical leadership had a significant negative effect on organizational dehumanization (*β* = −0.139, *p* < 0.001), indicating that paradoxical leadership significantly moderated the relationship between enhanced human-AI interaction and organizational dehumanization. As further illustrated by the simple slope analysis in Figure 2, the negative effect of enhanced human-AI interaction on organizational dehumanization was stronger when paradoxical leadership was high and weaker when paradoxical leadership was low. Therefore, H3a was supported.

**Figure 3:**
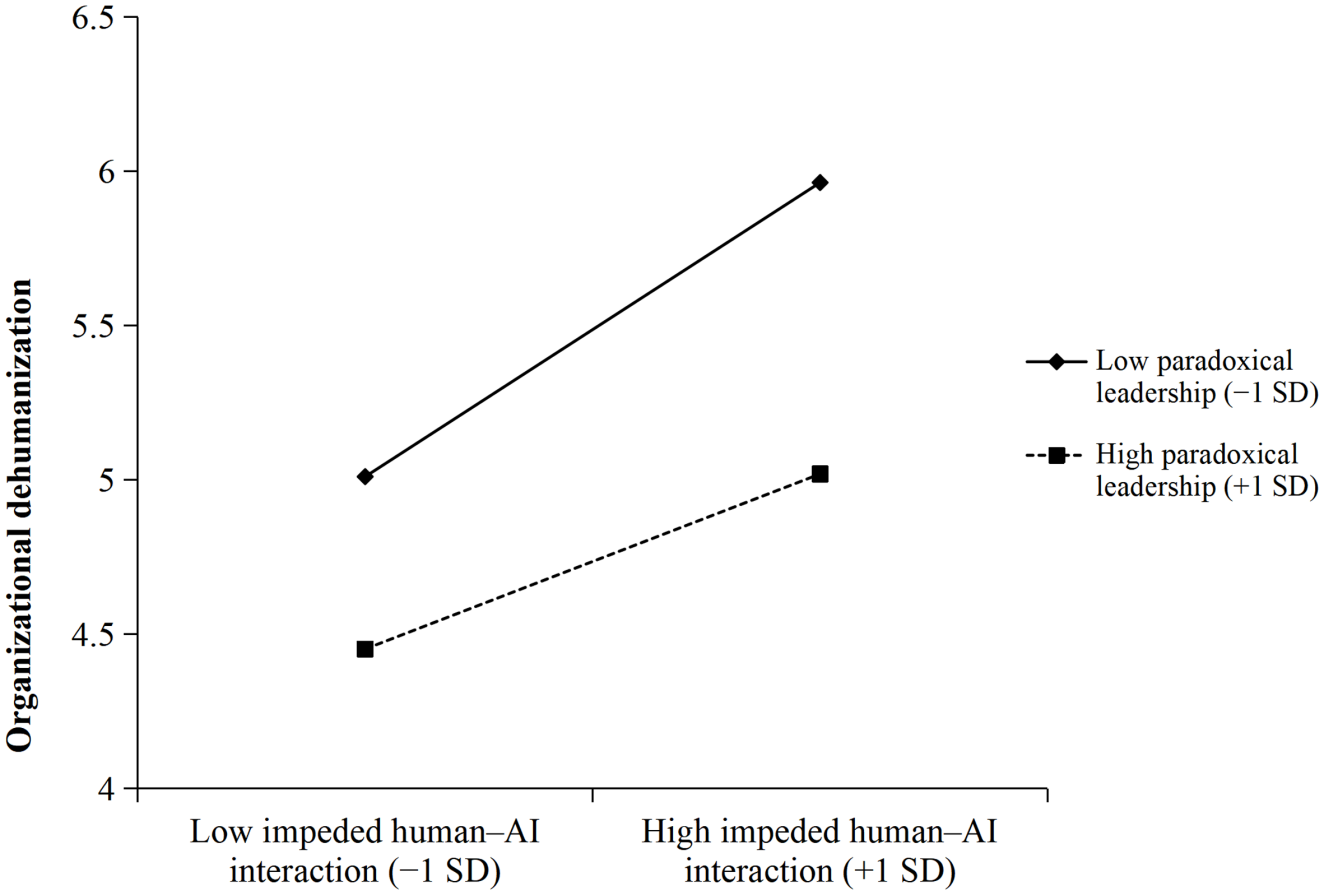
The moderating role of paradoxical leadership in the link between impeded Human-AI interaction and organizational dehumanization.

At the same time, the interaction between impeded human-AI interaction and paradoxical leadership also had a significant negative effect on organizational dehumanization (β = −0.207, p < 0.001). This finding indicates that as the level of paradoxical leadership increased, the positive effect of impeded human-AI interaction on organizational dehumanization became weaker. As shown by the simple slope analysis in Figure 3, the positive effect of impeded human-AI interaction on organizational dehumanization was stronger when paradoxical leadership was low, whereas this positive effect was significantly attenuated when paradoxical leadership was high. Therefore, H3b was supported.

##### 5.2.4.4 Moderated mediation effects

To further examine whether paradoxical leadership moderates the mediating process through which organizational dehumanization links enhanced and impeded human-AI interaction to knowledge territorial behavior, we conducted a moderated mediation analysis using Hayes’s (2018) PROCESS macro Model 7 with 5,000 bootstrap resamples to estimate the conditional indirect effects at different levels of paradoxical leadership. As shown in Table 8, in the path from enhanced human-AI interaction to knowledge territorial behavior, paradoxical leadership significantly moderated the first-stage path. The index of moderated mediation was −0.047, with a 95% confidence interval of [−0.079, −0.019], which did not include zero, indicating that the moderated mediation effect was significant. Further analysis showed that when paradoxical leadership was high, the indirect effect of enhanced human-AI interaction on knowledge territorial behavior through reduced organizational dehumanization was stronger, whereas this indirect effect was relatively weaker when paradoxical leadership was low. Therefore, H4a was supported.

**Table 8:** Test results of mediating effect and moderated mediating effect.

Effect type	Path	Effect	BootSE	95% Boot CI
Mediation effect	EHI → OD → KT	−0.162	0.024	[−0.209, −0.117]
	IHI → OD → KT	0.161	0.024	[0.111, 0.209]
Index of moderated mediation	EHI path	−0.047	0.015	[−0.079, −0.019]
	IHI path	−0.057	0.014	[−0.086, −0.031]
Conditional indirect effect	EHI (low PL)	−0.087	0.024	[−0.134, −0.045]
	EHI (medium PL)	−0.138	0.022	[−0.185, −0.096]
	EHI (high PL)	−0.189	0.031	[−0.255, −0.133]
	IHI (low PL)	0.203	0.030	[0.142, 0.263]
	IHI (medium PL)	0.141	0.022	[0.101, 0.185]
	IHI (high PL)	0.079	0.023	[0.037, 0.125]

Likewise, in the path from impeded human-AI interaction to knowledge territorial behavior, paradoxical leadership also significantly moderated the first-stage path. The index of moderated mediation was −0.057, with a 95% confidence interval of [−0.086, −0.031], excluding zero, which again indicates a significant moderated mediation effect. Further results showed that when paradoxical leadership was high, the indirect effect of impeded human-AI interaction on knowledge territorial behavior through increased organizational dehumanization was weaker, whereas this indirect effect was stronger when paradoxical leadership was low. Therefore, H4b was supported.

## 6 Discussion

First, this study finds that human–AI interaction exerts differential effects on knowledge territorial behavior, suggesting that employees’ perceptions of technology may produce distinct behavioral outcomes. On the one hand, enhanced human-AI interaction frees employees from routine tasks and helps preserve valued psychological resources and prevent unnecessary resource loss, thereby significantly reducing employees’ knowledge territorial behavior. This finding is consistent with prior research suggesting that AI, when perceived as an empowering tool, can stimulate positive emotions and enhance intrinsic motivation (Shagirbasha and Sivakumaran, 2021; Wang et al., 2022). On the other hand, impeded human-AI interaction deprives employees of workplace autonomy, fragments and mechanizes their work, and intensifies resource depletion, which in turn triggers defensive knowledge territorial behavior. This result echoes prior studies showing that AI-based control can undermine individuals’ self-concept (Tang et al., 2023a,b), increase burnout (Kong et al., 2021), and heighten emotional exhaustion (Teng et al., 2024). Taken together, these findings not only enrich the literature on human–AI interaction and knowledge territorial behavior, but also offer practical implications for organizations seeking to design effective technological interventions in the digital era. By understanding and optimizing different forms of human–AI interaction, organizations may be better positioned to address knowledge territoriality.

Second, this study demonstrates that organizational dehumanization is an important mediating mechanism through which human–AI interaction influences knowledge territorial behavior. Although organizational dehumanization has helped scholars explain how organizational contexts shape employee attitudes and behaviors, research on its role in the process of human–AI interaction remains limited. Existing studies have shown that organizational dehumanization can induce covert deviant behaviors such as employee silence, knowledge hiding, and time theft (Ahmed and Khalid Khan, 2016; Sarwar et al., 2021; Stinglhamber et al., 2025), and may even trigger withdrawal and exit-oriented coping strategies (Demoulin et al., 2021). The present findings are highly consistent with these conclusions from the perspective of conservation of resources (COR) theory, in that knowledge territorial behavior, like these other responses, can be understood as a defensive strategy adopted by individuals to cope with resource deprivation and restore a sense of control. By introducing organizational dehumanization as a mechanism directly relevant to human–AI work settings, this study reveals how human–AI interaction affects employees’ knowledge territorial behavior through its influence on employees’ feelings of being dehumanized by the organization. These findings not only extend the literature on organizational dehumanization, but also provide a new perspective for understanding how workplace technology may shape employees’ sense of instrumentalization.

Third, this study confirms the moderating role of paradoxical leadership in the process through which human–AI interaction influences knowledge territorial behavior. Leadership style plays a critical role in shaping employees’ work behaviors and psychological states, and different leadership contexts may influence how employees respond to specific work environments and technologies. By incorporating paradoxical leadership as a moderator, this study offers a more comprehensive perspective for understanding the relationship between human–AI interaction and knowledge territorial behavior in the context of digital transformation and technological implementation. Existing research has suggested that paradoxical leadership can promote knowledge sharing and reduce knowledge hiding by facilitating exchange opportunities and processes (Lee et al., 2010). However, the role of specific leadership styles in addressing the tensions and challenges introduced by AI use has remained insufficiently understood. Drawing on COR theory’s resource investment principle (Bhargava and Pradhan, 2019; Hobfoll, 1989), the present study finds that paradoxical leadership, by emphasizing the acceptance and balancing of competing demands (Zhang, 2015), can foster a complex yet inclusive environment, such as one that simultaneously emphasizes control and autonomy, or standardized treatment and individual consideration. This distinctive leadership style is especially effective in alleviating employees’ organizational dehumanization in human–AI collaboration settings. These findings not only extend the literature on paradoxical leadership, but also offer practical insights into how organizations may optimize leadership interventions when introducing artificial intelligence into the workplace.

### 6.1 Theoretical implications

First, this study extends research on the antecedents of knowledge territorial behavior by introducing a human–AI interaction perspective. Existing research on digitally transformed work settings has often treated artificial intelligence or technology use as a broad contextual backdrop, or has examined the overall impact of technological intervention without differentiating the nature of the human–AI relationship. As a result, findings in the literature on human–AI collaboration have often been mixed or inconsistent. Building on prior work, this study distinguishes between two forms of human–AI interaction (Li et al., 2025) and draws on this distinction to examine how different forms of interaction shape knowledge territorial behavior. In doing so, it enriches and extends the literature on human–AI interaction within organizational research.

Second, this study broadens the antecedent literature on knowledge territorial behavior in a more specific sense by moving beyond traditional workplace settings. Prior research has largely focused on conventional antecedents such as leaders’ bottom-line mentality (Tan et al., 2024; Zhang et al., 2020), psychological ownership (Brown et al., 2005; Silva de Garcia et al., 2022), and low-trust environments (Brown et al., 2014a,b), while paying limited attention to the role of human–AI interaction. Existing studies suggest that knowledge marking and knowledge defense are maladaptive responses to threats to psychological ownership or low-trust conditions (Demerouti et al., 2001; Gardner et al., 2019; Peng, 2013; Xu and Li, 2022). When organizations adopt human–AI collaborative work arrangements, however, algorithms and intelligent technologies disrupt existing resource boundaries, potentially increasing the complexity of knowledge territorial behavior by creating new forms of maladaptive adjustment. By examining the effects of human–AI interaction on employees’ knowledge territorial behavior, this study extends antecedent research on knowledge territoriality to AI-enabled work contexts.

Third, this study contributes to the literature on paradoxical leadership. A substantial body of research has examined paradoxical leadership, and some studies have shown that it plays a positive role in helping employees cope with complex and competing demands. For example, prior research suggests that employees working under paradoxical leadership are more likely to use artificial intelligence for knowledge sharing (Hu et al., 2025). However, whether leaders can effectively resolve tensions and suppress defensive responses when employees face the instrumentalization pressure associated with impeded human-AI interaction remains underexplored. From the perspective of conservation of resources theory, the present study shows that paradoxical leadership generally functions as a positive contextual resource. Specifically, it can effectively alleviate the organizational dehumanization associated with human–AI interaction and thereby reduce employees’ knowledge territorial behavior. Accordingly, this study not only enriches boundary condition research on how human–AI interaction influences employees’ covert defensive behaviors, but also deepens our understanding of the effectiveness of paradoxical leadership in the digital era.

### 6.2 Practical implications

First, because different forms of human–AI interaction exert differential effects on knowledge territorial behavior, organizations should reduce employees’ knowledge territorial behavior through effective work design. The findings show that enhanced human-AI interaction helps reduce knowledge territorial behavior, whereas impeded human-AI interaction is more likely to intensify it. Specifically, organizations should strengthen employees’ technology adaptation training so that they can quickly learn how to use AI effectively and develop stronger perceptions of enhanced human-AI interaction in their work. In addition, work should be designed in such a way that employees retain responsibility for core decision-making and creative tasks, while repetitive and mechanical activities are delegated to AI. By appropriately leveraging employees’ agency and professional expertise, organizations may reduce the likelihood of knowledge territorial behavior.

Second, organizations should take effective steps to alleviate employees’ organizational dehumanization. Because the two forms of human–AI interaction differ significantly in their effects on organizational dehumanization, organizations need to identify the roots of employees’ feelings of being instrumentalized and address them with targeted interventions. For example, organizations should strengthen employee training and clearly define the evaluation criteria and division of responsibilities between humans and machines during the work process, so that employees can fully leverage the advantages of AI while reducing the antecedents of organizational dehumanization. Training should also help employees understand how to collaborate with intelligent systems and how such systems complement and enhance, rather than replace, their work capabilities. Moreover, organizations should communicate more actively with employees about the purpose and benefits of AI implementation, emphasizing that human intelligence and creativity remain irreplaceable. When employees understand that human–AI collaboration is intended to improve work efficiency and quality while allowing them to focus on higher value-added tasks, their motivation to engage in defensive knowledge territorial behavior is likely to weaken.

Third, organizations should adopt measures to enhance managers’ paradoxical leadership. Organizations can provide specialized leadership training, educational opportunities, and development programs to help managers continuously improve the skills and knowledge needed to handle complex and competing demands. Through these developmental opportunities, managers may learn how to balance algorithmic efficiency with individualized concern for employees, thereby strengthening their paradoxical leadership capability. In addition, organizations may use performance appraisal systems, promotion opportunities, and public recognition to incentivize managers. When managers perceive that their efforts and effectiveness in balancing technological control with employee autonomy are recognized and rewarded by the organization, they are more likely to display paradoxical leadership behaviors, thereby providing stronger contextual resource support for employees and mitigating the knowledge-defensive tendencies triggered by technological disruption.

### 6.3 Limitations and future research directions

First, this study is limited by its research design and data sources. Study 2 still relied primarily on employees’ self-reported questionnaire data. Although self-reported measures are commonly used and considered appropriate for assessing knowledge territorial behavior—given that such defensive actions often involve covert psychological ownership and hidden intentions that are difficult for others to observe (Song and Cao, 2015; Yang et al., 2024)—this approach remains susceptible to social desirability bias and common method variance. Although the data were collected in two waves, this design cannot fully establish strict causal relationships. Future research could adopt multi-source data, such as ratings from coworkers or supervisors, or incorporate objective behavioral metrics, as well as multi-wave longitudinal designs, to test more rigorously the causal effects of human–AI interaction on employee behavior.

Second, the industry context of Study 2 may limit the generalizability of our findings. The sample was drawn from high-technology firms engaged in new-materials research and development, where proprietary knowledge, intellectual property boundaries, and confidentiality requirements are particularly salient. In such R&D-intensive settings, some forms of knowledge protection may be shaped by organizational mandates, formal confidentiality rules, or intellectual property policies rather than purely by employees’ individual psychological defense mechanisms. This possibility is especially relevant because firms in new-materials research and development often establish strict boundaries around technical knowledge, research data, and innovation-related information. Although our measure of knowledge territorial behavior focuses on employees’ discretionary tendency to mark, claim, and defend knowledge boundaries in intraorganizational interactions, we cannot fully rule out the possibility that industry-specific confidentiality norms influenced employees’ responses. Future research should replicate our model in less IP-intensive industries and directly control for confidentiality climate, IP protection norms, and formal knowledge-protection requirements. Such research would help further distinguish organization-mandated knowledge protection from psychologically driven knowledge territorial behavior and clarify the boundary conditions of our findings.

Third, this study is limited by its dimensional conceptualization of human–AI interaction. Specifically, we classified human–AI interaction into enhanced and impeded forms based on employees’ perceived functional experience of whether AI involvement facilitates or obstructs task completion, workflow efficiency, and resource preservation, rather than solely on differences in dominance and control. However, as intelligent technologies continue to evolve, the features of human–AI interaction are becoming increasingly complex. Future studies could move beyond a narrowly control-centered perspective and incorporate additional dimensions, such as the degree of AI anthropomorphism and algorithmic explainability, while also examining their effects on employees’ workplace behavior and possible spillover effects into nonwork domains.

Fourth, this study is limited by its relatively static perspective and its restricted consideration of contingency mechanisms. In particular, we did not capture employees’ dynamic adaptation process in response to technological disruption. Future research could adopt experience sampling or longitudinal approaches to trace the dynamic evolution of employees’ psychological resources as they become more familiar with technology over time. In addition, future studies could introduce variables such as knowledge-sharing climate and individual AI anxiety to more comprehensively extend the boundary conditions of the relationship between human–AI interaction and knowledge territorial behavior.

Fifth, this study is limited regarding its external validity and generalizability. Both studies were conducted using samples exclusively from high-tech firms in China. Employees in the high-tech sector typically possess higher AI literacy and may perceive technological disruption differently than workers in traditional manufacturing or service industries. Furthermore, Chinese cultural characteristics, such as collectivism and a high power distance orientation, might specifically shape how employees interpret leadership behaviors and enact territorial defense. Therefore, future research should endeavor to replicate our model across diverse industrial settings and different cultural contexts (e.g., Western countries) to verify and broaden the external validity of our findings.

Sixth, although our findings support the buffering role of paradoxical leadership, we acknowledge that paradoxical leadership may not always reduce the negative effects of impeded human–AI interaction. When leaders fail to integrate competing demands or when employees perceive paradoxical leadership as inconsistent control rather than balanced guidance, such leadership may increase role ambiguity and cognitive overload. Future research could further examine the conditions under which paradoxical leadership shifts from a contextual resource to an additional source of strain in AI-enabled workplaces.

Finally, although this study draws on COR theory to explain how human–AI interaction shapes employees’ psychological resource states and knowledge territorial behavior, we did not directly test a full resource gain spiral. Our mediator, organizational dehumanization, captures the reduction of a negative psychological state rather than the sequential accumulation of positive resources over time. Future research could examine genuine resource gain spirals in human–AI collaboration by adopting multi-wave longitudinal designs and incorporating positive mediators such as psychological empowerment, self-efficacy, work engagement, or perceived professional growth. Such research would provide a more direct test of whether initial resource gains from enhanced human–AI interaction enable employees to acquire additional positive resources over time.

## 7 Conclusion

This study examined the mechanisms through which human–AI interaction influences employees’ knowledge territorial behavior in the digital era. Building on the existing literature, we differentiated human–AI interaction into two dimensions, namely enhanced human-AI interaction and impeded human-AI interaction. Drawing on conservation of resources theory, we then conducted empirical analyses to investigate the double-edged pathways through which different forms of human–AI interaction shape knowledge territorial behavior, while systematically testing the mediating role of organizational dehumanization and the moderating role of paradoxical leadership. The findings not only enrich the literature on the antecedents of knowledge territorial behavior from the emerging perspective of human–AI collaboration, but also extend the effectiveness boundary conditions of paradoxical leadership. More importantly, they provide clear managerial guidance for organizations seeking to implement targeted leadership interventions and effectively prevent employees’ covert knowledge-defensive behaviors during digital transformation.

## Data Availability

The raw data supporting the conclusions of this article will be made available by the authors, without undue reservation.
